# Cefiderocol (Fetroja) as a Treatment for Hospital-Acquired Pneumonia

**DOI:** 10.7759/cureus.52230

**Published:** 2024-01-13

**Authors:** Connor J Plaisance, Grant E Borne, Charles P Daniel, Maxwell J Wagner, Anitha Shelvan, Jibin Mathew, Shahab Ahmadzadeh, Antonella Paladini, Giustino Varrassi, Sahar Shekoohi, Alan D Kaye

**Affiliations:** 1 School of Medicine, Louisiana State University Health Sciences Center, Shreveport, USA; 2 Department of Anesthesiology, Louisiana State University Health Sciences Center, Shreveport, USA; 3 Department of Life, Health & Environmental Sciences (MESVA), University of L'Aquila, L'Aquila, ITA; 4 Pain Medicine, Paolo Procacci Foundation, Rome, ITA

**Keywords:** resistance, pneumonia, carbapenemase, gram-negative bacilli, siderophore, cefiderocol

## Abstract

With increasing resistance to conventional antibiotic treatments, especially among gram-negative bacilli, the search for new antibiotics has become critical on a global scale. Among infections with multidrug-resistant bacteria is hospital-acquired pneumonia (HAP), which is nosocomial pneumonia in patients who have been hospitalized for more than 48 hours. HAP carries a high mortality rate and continues to be a challenge with regard to adequate treatment. The typical multidrug-resistant gram negatives found in HAP include *Pseudomonas aeruginosa*,* Klebsiella pneumoniae*, and *Acinetobacter baumannii*. Many new antibiotics have been studied and tested against these pathogens as possible solutions, and the search continues. Cefiderocol, a novel siderophore cephalosporin, is effective against these pathogens. Cefiderocol is an iron-chelating agent that makes use of iron pumps on the membrane of bacteria via a catechol moiety on the C3 side chain of the molecule. This allows for easy access into the cytoplasm, where it can inhibit peptidoglycan synthesis by binding to penicillin-binding proteins. Cefiderocol displays linear pharmacokinetics and is mainly excreted through the kidneys. It is well tolerated in healthy individuals but may need adjustments of dosage in patients with impaired renal function. Studies have shown that both healthy subjects and those with impaired renal function experienced some adverse effects, including nausea, diarrhea, abdominal pain, and increased creatinine kinase; however, these adverse effects were limited and experienced in placebo groups. It has demonstrated efficacy in treating infections caused by many multidrug-resistant gram-negative pathogens and has demonstrated high stability against many classes of b-lactamases. There have been multiple phase 3 trials, such as the CREDIBLE-CR trial and the APEKS-NP trial, that demonstrated efficacy in treated nosocomial pneumonia caused by multidrug-resistant gram negatives, such as carbapenem-resistant *Acinetobacter baumannii*, *Klebsiella pneumoniae*, and *Pseudomonas aeruginosa*, compared to the best available treatment. While clinical data remain limited, a few studies are showing clinical efficacy and few adverse effects. Cefiderocol demonstrated effectivity in treating multidrug-resistant gram-negative pneumonia in patients with multiple comorbidities, such as chronic kidney disease, chronic-obstructive pulmonary disease, and diabetes mellitus. Cefiderocol shows promise as a novel antimicrobial agent in treating multidrug-resistant gram-negative in HAP.

## Introduction and background

Adequate and effective treatment for hospital-acquired pneumonia (HAP) has become an issue of utmost importance as it carries high morbidity and mortality rates. Increasing cases caused by multidrug-resistant (MDR) gram-negative bacteria are an issue on a global scale. HAP is defined as pneumonia that is acquired by patients after at least 48 hours of hospitalization. A subset of HAP, ventilator-associated pneumonia (VAP), is pneumonia acquired by patients placed on a mechanical ventilator [1]. Many microbial culprits cause HAP, with the majority of the cases being caused by gram-negative bacilli. Viruses have become increasingly implicated as causes of HAP. The typical gram-negative bacilli indicated in HAP include *Klebsiella pneumonia*, *Psuedomonas aeruginosa*, and *Acinetobacter baumannii*. Gram positives are also implicated in HAP and include *Staphylococcus aureus* and *Streptococcus pneumoniae*. Less common pathogens include *Mycobacterium tuberculosis*, non-tuberculosis mycobacteria, *Chlamydophila* spp., *Coxiella*, and fungi, such as *Coccidiodes*, *Blastomycoses, Histoplasma, Cryptococcus, and Aspergillas*. Many factors play a role in the presence of multidrug resistance, including recent antibiotic use, comorbidities such as chronic obstructive pulmonary disease (COPD), and length of hospitalization [1,2]. With the emergence of new MDR and carbapenem-resistant gram-negative bacilli, many studies have delved into different antibiotics that show promise in controlling and eliminating HAP [3-5]. The World Health Organization (WHO) has designated carbapenem-resistant *Enterobacteriaceae*, *Pseudomonas aeruginosa*, and *Acinetobacter baumannii* as high-priority pathogens for which new antibiotic treatments are needed urgently [6].

Several novel antibiotics have been studied and are effective in limiting the expansion of antimicrobial resistance. These antibiotics include ceftazidime-avibactam, meropenem-vaborbactam, imipenem-cilastatin-relebactam, plazomicin, and eravacycline. However, even with these new and approved antibiotics, there are still difficulties encountered in treating gram-negative bacilli that express *Klebsiella pneumoniae* carbapenemase (KPC), oxacillin (OXA) carbapenemase, and metallo-b-carbapenemases in particular [3,7,8]. Cefiderocol, a novel siderophore cephalosporin, has shown promise as a potential antimicrobial agent to treat HAP, specifically those caused by MDR and carbapenem-resistant gram-negative bacilli. Many studies in human and mouse models have shown efficacy in treating gram-negative bacilli exhibiting the difficult-to-treat multidrug resistance factors, including KPC, oxacillin carbapenemase, metallo-b-lactamase, and extended-spectrum beta-lactamase. Cefiderocol displays similar molecular features as ceftazidime and cefepime, which are third- and fourth-generation cephalosporins, respectively. However, cefiderocol displays a unique catechol moiety on the C3 position of the side chain, which allows entry into bacteria via iron chelation. Cefiderocol has demonstrated equal or superior ability compared to ceftazidime-avibactam in the treatment of *Acinetobacter baumannii*, KPC-producing *Enterobacteriacae*, and *Pseudomonas aeruginosa* due to its high stability to b-lactamases [9,10]. With its superior ability to eliminate infections of MDR and carbapenemase non-susceptible gram-negative bacilli, as well as its favorable pharmacokinetic profile, cefiderocol shows promise as a novel antimicrobial that can be used to decrease HAP mortality.

## Review

HAP-associated factors

HAP is characterized as pneumonia that develops in a patient who has been hospitalized for a minimum of 48 hours, residing in a nursing home or extended care facility, or receiving chronic therapies, such as dialysis. VAP, a subtype of HAP, is described as pneumonia that develops in a patient 48-72 hours following endotracheal intubation [11,12]. The incidence of HAP ranges from five to more than 20 cases per 1,000 hospitalizations [13]. HAP is the second most common nosocomial infection globally and the most common cause of death in nosocomial infections. Infection most often occurs in patients who are immunocompromised, surgical patients, and the elderly. HAP extends hospital stays by an average of seven to nine days and costs more than $40,000 in hospital bills per patient [13]. Sources of infection for HAP can vary. However, many examples include healthcare devices, the hospital environment, and the transfer of pathogens unknowingly from staff to patients [13]. Aspiration has been identified as an important factor in the development of both HAP and VAP. Medications that suppress gastric acid production, such as proton-pump inhibitors and histamine-2 receptor blockers, increase the ability of pathogens to colonize the oropharynx and be aspirated [11].

While our understanding, as well as the protocols to treat HAP and VAP, have become more robust, the mortality associated with these infections remains a serious issue. There are many challenges to diagnosing HAP, as there are many factors that can contribute to ICU patients requiring more oxygen and developing leukocytosis. Declining respiratory status in a patient, fever, and cough can be signs of developing pneumonia. According to the Infectious Diseases Society of America/American Thoracic Society (IDSA/ATS) guidelines, a diagnosis of HAP requires the following criteria: new infiltrates on chest imaging, respiratory decline, fever, and productive cough [13]. Both HAP and VAP are associated with a mortality rate that can range anywhere from 20% to 50%, even reaching 75% in instances when the causative agent is an MDR pathogen [14-16]. Studies have shown that when HAP is caused by MDR bacteria, the mortality rate is higher, possibly due to the delay in the initiation of adequate antibiotic treatment [17]. These pathogens include *P. aeruginosa*, *K. pneumonia*, *Acinetobacter* spp., and *Enterobacteriaceae*. Gram-positive pathogens include *S. aureus*, in particular methicillin-resistant *S. aureus* (MRSA). The period in which a patient develops HAP during their hospitalization has been linked to outcomes and is considered a modifiable risk factor. HAP that develops early during a patient's hospitalization (zero to four days) correlates with better outcomes as the pathogens involved are typically more sensitive to treatment. Infections that develop after five days tend to be caused by MDR bacteria and are associated with higher morbidity and mortality [13,18].

MDR pathogens in HAP

Many different species of bacteria cause HAP, along with other less common causes, such as fungi and viruses in immunocompromised individuals. There is an increasing incidence of HAP as a result of infection by MDR pathogens. These include many aerobic gram-negative bacilli, such as *P. aeruginosa*, *K. pneumonia*, and *Acinetobacter* spp. *Enterobacter* species express patterns of hospital-acquired resistance due to a shared chromosomal AmpC beta-lactamase that is highly expressed due to mutation and confers resistance to beta-lactase and alpha-methoxy-beta-lactams, including cefoxitin and cefotetan. Gram-positive pathogens include *S. aureus*, in particular methicillin-resistant *S. aureus* [13]. *S. aureus* is a more common cause of pneumonia in individuals who have diabetes or head trauma or are hospitalized in the ICU. These pathogens account for around 80% of all HAP [13,19].

*Pseudomonas*, the most common of the MDR pathogens implicated in HAP, has intrinsic resistance to many antibiotics due to its use of efflux pumps. These pumps are called multidrug efflux pumps (Mex). In the United States, *Pseudomonas* resistance is on the rise due to many antibiotics, including piperacillin, cephalosporins, carbapenems, aminoglycosides, and fluoroquinolones [13,20]. Resistance to antibiotics, such as piperacillin, ceftazidime, cefepime, other oxyimino-β-lactams, imipenem and meropenem, aminoglycosides, or fluoroquinolonesis, has become an increasing issue. *Pseudomonas* can become resistant to carbapenems, such as meropenem or imipenem, based on the expression of an outer membrane porin channel (OrpD).

*Klebsiella *species carry intrinsic resistance ampicillin and can acquire resistance to cephalosporins through the production of extended-spectrum b-lactamases (ESBLs) [13]. *Klebsiella* species that produce EBSLs typically remain susceptible to carbapenems. However, similar to *Pseudomonas*, the loss of an outer membrane porin channel can lead to the development of resistance. 

*Acinetobacter *species display resistance through the production of carbapenemases, including oxacillin (OXA)-carbapenemases [13]. Studies have shown that 85% of *Acinetobacter* strains are susceptible to carbapenems, but resistance is nonetheless increasing. With the rise in resistance to many widely used and available antibiotics, many novel antibiotics have been studied in recent years as potential solutions to the ever-growing antibiotic resistance issue. These include, but are not limited to, ceftobiprole, ceftolozane-tazobactam, merepenem-vaborbactam, imipenem-relebactam, and cefiderocol [l2]. Sulbactam can also be used as an alternate therapy [13]. 

MRSA accounts for about 50% of ICU infections and is another pathogen of multidrug resistance that is becoming harder and harder to treat. MRSA exhibits its resistance by producing penicillin-binding proteins (PBPs) that have less affinity for beta-lactams, which are encoded by the *mecA *gene [13]. MRSA is resistant to the entirety of beta-lactam antibiotics, with vancomycin being the main treatment option. 

Mechanism of action of cefiderocol

Cefiderocol is a novel siderophore cephalosporin that has an antimicrobial activity against gram-negative bacilli, including *Enterobacterales *that produce many different carbapenemases and difficult-to-treat non-fermenters. The wide spectrum of coverage displayed by cefiderocol is due to its high stability against all classes of b-lactamases, including Ambler classes A, B, C, and D, and extended-spectrum b-lactamases. This stability to b-lactamases is likely due to the properties of the C3 and C7 side chains. Cefiderocol has structural similarities to both cefepime and ceftazidime. Regarding cefepime, it carries a similar pyrrolidinium group on the C3 side chain, which enhances antimicrobial activity and stability to b-lactamases. It also carries an aminothiazole ring and a carboxypropyl-oxyimino group on the C7 side chain, similar to cetazidime. However, it contains a unique catechol moiety on the C3 position of the side chain that promotes transport into cells via the chelation of ferric iron and accessing iron pumps on the bacterial cell membrane, such as the pneumococcal iron uptake protein A (PiuA) in *P. aeruginosa* [10]. This facilitates entry into the bacteria and accumulation in the periplasmic space. This allows it to evade many of the antibiotic resistance mechanisms, such as porins, efflux pumps, and b-lactamases. Cefiderocol exerts its effects by binding to penicillin-binding protein 3 (PBP-3) and penicillin-binding protein 2 (PBP-2), which inhibit the synthesis of peptidoglycan, leading to cell death [7,9].

Cefiderocol susceptibility

The SIDERO-WT studies were studies conducted since 2014 on over 28,000 gram-negative isolates and their susceptibility to cefiderocol. This was a large surveillance study conducted on 8,954 isolates from North America and Europe to determine susceptibility to cefiderocol compared to other commonly used antibiotics. These isolates included *Enterobacterales* (*E. coli*, *Klebsiella* spp., *Citrobacter* spp., and *Enterobacter* spp.,), *P. aeruginosa*, and *A. baumannii*. The minimum inhibitory concentration against 90% of the isolates tested (MIC90) for *Enterobacterales* ranged from 0.25 to 1 mg/L. Surveillance studies on the *Enterobacterales* isolates demonstrated that >98% and 99% of isolates were inhibited at 2 mg/L and 4 mg/L, respectively. The study demonstrated that cefiderocol maintained activity against isolates that were resistant to extended-spectrum cephalosporins and carbapenems. The MIC90 values for *P. aeruginosa* were 0.5-2 mg/L, with cefiderocol showing an inhibitory capacity of >99% at 4 mg/L. It also demonstrated activity against *P. aeruginosa* isolates that were resistant to ceftolozane/tazobactam. This was demonstrated at >98% of isolates inhibited at 2 mg/L. Against *A. baumannii*, it showed to have an *MIC90 *of 1-2 mg/L and inhibited >95% of isolates at 4 mg/L as well. This included *Acinetobacter *species that produced OXA-carbapenemases [21].

Cefiderocol pharmacokinetics and safety profile

The pharmacokinetic and safety profiles of cefiderocol have been assessed extensively in both healthy subjects and subjects with renal impairment. Katsube et al. conducted a non-randomized, open-label cohort study comparing the pharmacokinetics of cefiderocol in subjects with varying degrees of renal impairment compared to a healthy control group. All subjects received an intravenous infusion of 1,000 mg over the course of an hour, with the subjects requiring hemodialysis (HD) receiving one dose one hour after HD and another dose two hours before their next scheduled HD, with a 72-hour washout period in between. In cases with differing levels of renal dysfunction, being mild, moderate, or severe, the pharmacokinetics of cefiderocol varied. The area under the curve (AUC) for the plasma concentration-time curve was increased with decreasing renal function. This indicates that the dosage for patients with renal dysfunction may need to be adjusted to optimize efficacy. This could be achieved by reducing the dosage given or increasing the dosing interval to avoid drug accumulation. The plasma-protein-unbound fraction was measured in groups of patients with varying levels of renal function and was found to be similar among all the groups. The study also found that cefiderocol was significantly removed via intermittent hemodialysis in patients with severe renal dysfunction. The only adverse effects reported for cefiderocol in the study on the antibiotic's effect on patients with renal impairment included urticaria, contact dermatitis, and nausea in the cohorts with renal impairment. Only one patient in the healthy subject cohort experienced myalgia. These adverse effects were only reported by half or fewer subjects in each cohort. There were no significant changes in the physical exam, vital signs, 12-lead ECG, or laboratory studies (hematology, blood chemistry, and urinalysis) [22].

Cefiderocol displays linear pharmacokinetic behavior with a mean half-life of 1.98-2.74 hours, with the mean total clearance being 4.6-6.0 L/hr in healthy subjects. The dosages in the study ranged from 100 mg to 2000 mg. Cefiderocol is excreted mainly through the kidneys, with the study finding 60%-70% of the parent drug in the urine. In the group of healthy volunteers receiving cefiderocol at a dosage of 2,000 mg every eight hours, there was no drug accumulation, and it was well tolerated over 10 days. It was also noted in phase I studies that a dose of up to 4000 mg and multiple 2000 mg doses were well tolerated in healthy subjects [23,24]. In both dosage groups, adverse effects, such as diarrhea, abdominal pain, nausea, rash, blood in urine, and an increase in white cells, were noted. The multiple dosage group also showed increased blood urea, creatinine kinase, and liver enzymes. However, these effects were also noted in the respective placebo groups. As cefiderocol is an iron-chelating antibiotic, studies on blood iron were conducted in subjects taking cefiderocol. There were no significant effects on iron noted [23].

In a thorough evaluation of cefiderocol on the QT interval, using moxifloxacin as in the placebo group, it was shown that cefiderocol has no clinically significant effects on the QT interval [24].

Cefiderocol in the treatment of hospital-acquired bacterial pneumonia

Currently, there are few approved antibacterial drugs used in the treatment of hospital-acquired and ventilator-associated bacterial pneumonia, and cefiderocol is one of the most promising of the approved drugs. The approved drugs include ceftobiprole, ceftolozane-tazobactam, meropenem-vabrobactam, imipenem-relebactam, and cefiderocol [2]. Cefiderocol’s usefulness as compared to other drugs of this class is due to its ability to treat pathogens that are often MDR. This characteristic of cefiderocol is of increasing importance as pathogens are rapidly mutating and finding ways to become untreatable with current antibiotic regimens. Cefiderocol is especially effective against gram-negative bacteria [25]. Some of the species that cefiderocol is effective against in a laboratory environment include *Enterobacteriaceae*, *P. aeruginosa*, and *A. baumannii* [21]. Cefiderocol’s ability to treat these species is crucial because they are resistant to many other antibiotics, including carbapenems. The structure of cefiderocol allows it to overcome the defenses of many microbial species, such as efflux pump expression and cessation of expression of porin channels [26].

The ability of cefiderocol to effectively treat a variety of bacterial organisms that commonly cause HAP in a clinical setting is heavily reliant on it being safe to treat patients with. While the phase 3 trials for the drug did indicate an elevation in liver enzymes for some study participants, they did not reach levels indicative of serious liver injury, but recommendations have been made to monitor liver enzymes for patients treated with cefiderocol [26].

When considering new treatments for a pathology as common and devastating as HAP, it is important to establish a new treatment as effective as the regimen currently in use. In multiple studies, cefiderocol has been proven to be comparable to currently approved drugs. The APEKS-NP study was conducted to evaluate the efficacy of cefiderocol in the treatment of hospital-acquired and ventilator-associated pneumonia compared to the conventional treatment, meropenem. The study was a randomized, double-blind, parallel-group, phase 3, non-inferiority trial that took place in 17 different countries across Asia, Europe, and the United States. Adults aged 18 and older with hospital-acquired or ventilator-associated pneumonia were enrolled and randomly assigned to either the cefiderocol or meropenem group. Each group was given a three-hour infusion of 2,000 mg of either cefiderocol or meropenem every eight hours for seven to 14 days. In addition, patients received intravenous linezolid at 600 mg/five hours for at least five days. The basis of the study was to determine the all-cause mortality in the intention-to-treat population between cefiderocol and meropenem. The gram-negative pathogens that were the causative agents in the patients studied included *Klebsiella pneumoniae*, *P. aeruginosa*, *A. baumannii*, and *Escherichia coli*. The all-cause mortality at day 14 for the cefiderocol population was 12.4%, while the all-cause mortality in the meropenem population was 11.6%. This study demonstrated that cefiderocol was non-inferior to meropenem in terms of all-cause mortality on day 14 in patients with gram-negative hospital-acquired or ventilator-associated pneumonia, suggesting cefiderocol as a potential treatment for these infections [27].

While there have been many studies touting the promise of cefiderocol as a treatment for hospital-acquired bacterial pneumonia, there has been a study determining *Acinetobacter* as a possibly troublesome species for the drug. The CREDIBLE-CR study was a randomized, open-label, multicenter, pathogen-focused phase 3 trial performed to determine the efficacy of cefiderocol versus the best available treatment in patients with carbapenem-resistant gram-negative infections. The study, like the APEKS-NP study, was done in 16 countries in North and South America, Asia, and Europe. Cases aged 18 or older who were diagnosed with nosocomial pneumonia, sepsis, or complicated urinary tract infection caused by carbapenem-resistant gram-negative bacteria. Patients received either a three-hour intravenous infusion of 2,000 mg of cefiderocol every eight hours or the best available treatment option for seven to 14 days. The endpoint of the study was clinical cure at the test of cure (7 ± 2 days after the end of treatment) in the carbapenem-resistant intention-to-treat population. The most frequent carbapenem-resistant gram-negative bacteria in the patients in the study were *A. baumannii*, *K. pneumoniae*, and *P. aeruginosa*. In patients with nosocomial pneumonia, the clinical cure was achieved by 20 out of 40 patients in the cefiderocol group and 10 out of 19 in the best available treatment population. In the population of patients with bloodstream infection/sepsis, the clinical cure was achieved in 10 out of 23 patients in the cefiderocol group and 6 out of 14 in the best available treatment population. For patients with complicated UTIs, the clinical cure was achieved in 9 out of 17 patients in the cefiderocol group and 1 out of patients in the best available treatment group. In the safety analysis, the study found that treatment-emergent adverse effects were noted in 91% of the cefiderocol group and 96% of the best available treatment group. The CREDIBLE-CR study determined that cefidercol was similar in efficacy compared to the best available treatment in the treatment of carbapenem-resistant gram-negative infections. However, the study also demonstrated that there were more deaths in the cefiderocol group in the treatment of infections caused by *Acinetobacter* spp. [28] (Table 1).

**Table 1:** Clinicals trials on the efficacy and safety of cefiderocol

Author (year)	Groups studied and intervention	Results and findings	Conclusions
Wunderink et al. [27] (2021)	The APEKS-NP study was a randomized, double-blind, parallel-group, phase 3, non-inferiority trial that took place in 17 different countries across Asia, Europe, and the United States. Adults aged 18 and older with hospital-acquired or ventilator-associated pneumonia were enrolled and randomly assigned to either the cefiderocol or meropenem group. Each group was given a three-hour infusion of 2,000 mg of either cefiderocol or meropenem every eight hours for seven to 14 days.	Mortality rate (%) Cefiderocol: 12.4% Meropenem: 11.6%	At the 14-day mark, the mortality rate of cefiderocol is not significantly higher than meropenem when used as a treatment for hospital-acquired pneumonia. This study proved that cefiderocol was not inferior to meropenem in the treatment of HAP/VAP.
Bassetti et al. [28] (2021)	The CREDIBLE-CR study was a randomized, open-label, multicenter, pathogen-focused phase 3 trial performed to determine the efficacy of cefiderocol versus the best available treatment in patients with carbapenem-resistant gram-negative infections. Patients aged 18 or older who were diagnosed with a nosocomial pneumonia, sepsis, or complicated urinary tract infection caused by carbapenem-resistant gram-negatives. Patients received either a three-hour intravenous infusion of 2000 mg of cefiderocol every eight hours or the best available treatment option for seven to 14 days.	Clinical cure (%) Cefiderocol: 50% Best Available therapy: 53% Mortality rate (%) Cefiderocol: 34% Best Available therapy: 18%	When treating a gram-negative nosocomial pneumonia, cefiderocol has been shown to have a similar clinical cure rate compared to the best available treatment. However, a higher mortality rate was seen in cefiderocol groups in the treatment of *Acinetobacter* spp.
Falcone et al. [29] (2022)	This was an observational retrospective study on patients who were diagnosed with bloodstream infections/sepsis, ventilator-associated pneumonia, or other infections due to carbapenem-resistant* Acinetobacter baumannii* and were divided into two groups based on whether they received cefiderocol or colistin regimens. The endpoint of the study was a 30-day mortality between the two groups.	30-day mortality rate Cefiderocol group: 34% Colistin group: 55.8%	Cefiderocol shows promise in treating severe carbapenem-resistant *Acinetobacter baumannii;* however, a clinical trial is likely needed to confirm.

Kresken et al. performed a study on the effects of cefiderocol on 217 sets of random clinical gram-negative bacterial isolates in Germany. These isolates included 17 extended-spectrum beta-lactamase-producing bacteria, 13 isolates of *A. baumannii*, and 54 isolates of *P. aeruginosa*. There were also isolates of various challenge bacteria that produce MDR proteins, such as carbapenemases. These isolates were included. The antibiotics that cefiderocol was compared to in the study included (with their concentration ranges noted in parenthesis following each one) amikacin (4-64 mg/L), aztreonam (0.5-32 mg/L), cefepime (0.5-16 mg/L), cefiderocol (0.03-64 mg/L), ceftazidime (0.03-64 mg/L), ceftazidime-avibactam (0.03/4-64/4 mg/L), ceftolozane-tazobactam (0.03/4-64/4 mg/L), ciprofloxacin (0.25-4 mg/L), colistin (0.5-8 mg/L), and meropenem (0.03-64 mg/L). The minimum inhibitory concentration was determined using micro-dilution, and an iron-depleted medium was used. The study showed that for the 146 *Enterobacterales* isolates tested, 95.9% were inhibited by cefidercol MIC50 and MIC90 concentrations of 0.12 and 0.1 mg/L, respectively. Of the 13 *A. baumannii *isolates tested, cefiderocol inhibited all isolates at concentrations of <0.12 mg/L. Of the 54 *P. aeruginosa* isolates tested, cefiderocol inhibited 100% of isolates at ≤1 mg/L. It was shown to be more active than ceftolozane-tazobactam, ceftazidime-avibactam, or meropenem. This is another example of a study showing promise in cefiderocol as a novel agent in the treatment of MDR gram-negative pathogens that are commonly implicated in HAP compared side by side to current treatments [3].

There are limited data on cefiderocol used in clinical settings, but the studies that have been performed in a clinical setting demonstrate that cefiderocol is effective in treating pneumonia caused by MDR pathogens. Studies show that cefiderocol demonstrates a survival rate of up to 70-90% in patients with pneumonia and bloodstream infections and those in ICU [29,30,31,32]. Kurrali et al. conducted an observational, retrospective, single-center clinical study on the effectiveness of cefiderocol in patients with pneumonia caused by MDR gram-negative rods. The study consisted of 28 patients who had comorbidities, such as prior myocardial infarction, chronic kidney disease, diabetes mellitus and COPD, solid cancer, heart failure, and peripheral vascular disease. The main pathogens in these individuals were found to be *Acinetobacter* spp., *Pseudomonas* spp., and *E. coli*. They found that cefiderocol proved to be effective, with an overall therapeutic success rate (improvement/resolution of the disease) of 64.3% at seven days and 50% at 14 days from the start of treatment. They did not report any adverse events associated with cefiderocol treatment [31]. 

Recent studies have discovered that there is however increasing cefiderocol resistance. Karakonstantis et al. performed a systematic review that included 52 studies that were completed in 2018. According to their research, resistance to cefiderocol has increased in recent cohorts and is due to multiple mechanisms. Mutations in β-lactamases, porins, siderophore receptors, efflux pumps, and penicillin-binding proteins are some of the main ways that resistance to cefiderocol has grown, specifically co-expression of multiple β-lactamases, often in combination with permeability defects. They also discovered that one or more of these alterations lead to a higher baseline cefiderocol MIC, resulting in resistance. The usual culprit for resistance is normally not only one of these alterations but rather a combination [33].

Discussion

HAP remains one of the primary causes of mortality in patients with a nosocomial infection, and the mortality rate among patients with HAP remains very high. With the ever-growing issue of antibiotic resistance and the evolution of MDR bacteria as the infectious etiology of HAP, the clock is ticking to find antibiotics and therapies that can counter and eliminate these infections. The data are clear in showing that the longer it takes to identify and appropriately treat the infectious agent in HAP, the worse the outcome is for the patient. The growing issue of multidrug resistance has brought on a new wave of antimicrobial agents that hold promise in combating these infections, which will continue to be assessed in clinical trials to determine and confirm their efficacy as first-line treatments for MDR gram-negative infections. Cefiderocol holds promise as a new antimicrobial in the treatment of MDR infections, in particular HAP. The increase in infections caused by difficult-to-treat pathogens with resistance to many conventional therapies remains an issue of utmost importance on a global scale. Among the many novel antimicrobial agents studied as potential solutions to the ever-growing multidrug resistance issue, cefiderocol displays many favorable attributes that make it a promising solution for the future. With its broad-spectrum coverage, high stability against b-lactamases, favorable pharmacokinetic and safety profile, and efficacy against pathogens, such as *P. aeruginosa*, *K. pneumoniae*, and *A. baumannii*, it shows promise as one of the many novel antimicrobials that can eliminate HAP and ventilator-associated pneumonia caused by MDR gram negatives [30].

Even with the data that are currently published on cefiderocol, there are still some limitations to the current literature. For example, the CREDIBLE-CR study used a small sample size and heterogenous patient population, which therefore may have limited the number of factors for stratification and subsequently hindered randomization. In the future, studies should contain larger sample sizes to increase randomization and identify any trends in adverse effects. Other factors should also be considered in the future, such as ICU admission at baseline or sepsis or septic shock before randomization to better balance the risk of all-cause mortality. Furthermore, studies on newly emerging heteroresistance should be looked into in the future as it has been reported but not very well studied. Heteroresistance is when a smaller population of resistant pathogens exists among a larger population of resistant pathogens, but the mechanism of resistance among each population differs. For example, Choby et al. took a look into the CREDIBLE-CR trial and investigated why cefiderocol leads to a higher all-cause mortality compared to the standard treatment, in particular *Acinetobacter* spp. infections. They hypothesized this could be due to emerging heteroresistance. They found that the frequency of heteroresistance was far greater than that of detected resistance for all four pathogens and was similar to the all-cause mortality rate, suggesting that heteroresistance might have contributed to the increase in all-cause mortality in the CREDIBLE-CR study [28,34]. 

The data concerning the cost effectiveness of cefiderocol are limited. However, a study by Yahav et al. analyzed the cost effectiveness of cefiderocol in comparison to other "new" antibiotics (noted to be antibiotics created after 2010 in this particular study) and "old" antibiotics (noted to be those antibiotics created before 2010 in this particular study). They found that, particularly in the treatment of carbapenem-resistant gram-negative infections, cefiderocol would cost the US about 150 million more USD annually compared to the use of colistin, which is about 20 times the cost of colistin annually. Similar increases in costs were also seen in infections being treated with ceftazidime-avibactam, ceftolozane-tazobactam, meropem-vaborbactam, and impanel-sulbactam [35]. According to the Fetroja Product Ordering Fact Sheet, the wholesale acquisition cost of a carton of 10 with 1 g vials of cefiderocol is $1,897.50. Shionogi, a Japanese pharmaceutical company, was granted a new technology add-on payment to help assist patients with the cost of cefiderocol, particularly those under Medicare. This new technology add-on payment provided patients with up to 75% reimbursement for the cost of cefiderocol treatment. The aforementioned data are relevant to and based on usage in the United States, but data may differ internationally. Bassetti et al. performed a study in Italy focusing on the cost effectiveness of cefiderocol vs. ceftazidime/avibactamin in the treatment of complicated urinary tract infections, sepsis, and pneumonia caused by carbapenem-resistant pathogens, such as *Enterobacterales*, *Acinetobacter* spp., *Stenotrophomonas *spp., and *Pseudomonas*. The study consisted of a decision-tree model comparing the cost effectiveness of cefiderocol. Results of the study show that cefiderocol is highly cost effective when compared against ceftazidime/avibactam in this patient population. The cost per patient was €11,017 and €9,538 for cefiderocol and ceftazidime/avibactam, respectively [36].

## Conclusions

There are still more trials and investigations that need to be performed with cefiderocol to ensure its clinical efficacy among other treatment options available for MDR gram-negatives. With the usage of cefiderocol, further investigation is warranted in the long-term safety profile of the drug, particularly in immunocompromised patients or those with comorbidities, such as chronic kidney disease, diabetes, and cardiovascular disease, to facilitate its use as a mainstay treatment for difficult-to-treat HAP. It would also be beneficial to include information concerning the regulation of cefiderocol use in the hospital and strategies to prevent improper use in future research. Cost analysis and effectiveness should be studied as they pose a potential financial and social barrier to access compared with cheaper and more conventional treatments in current use. However, it is difficult to overlook the efficacy that has already been proven in its ability to achieve clinical cure in MDR gram-negative infections when compared side by side to the best available treatments.
